# BSA Adsorption on Titanium Dioxide Nanoparticle Surfaces for Controlling Their Cellular Uptake in Skin Cells

**DOI:** 10.1021/acsabm.3c01138

**Published:** 2024-03-04

**Authors:** Raweewan Thiramanas, Yodsathorn Wongngam, Goragot Supanakorn, Duangporn Polpanich

**Affiliations:** National Nanotechnology Center (NANOTEC), National Science and Technology Development Agency (NSTDA), Pathum Thani 12120, Thailand

**Keywords:** titanium dioxide, bovine serum albumin, cellular uptake, functionalization, nanoparticle-based sunscreen

## Abstract

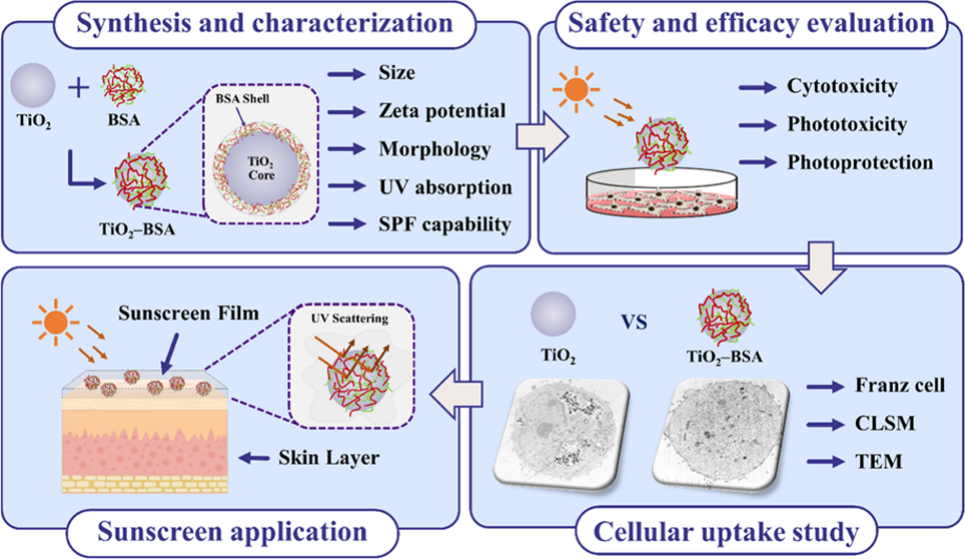

Nanoparticles (NPs) are continuously being developed for many applications including imaging, biomedicine, and everyday products. It is difficult to avoid contact with NPs such as titanium dioxide (TiO_2_) NPs, which are widely used in sunscreens. However, the safety of TiO_2_ NPs for skin contact and inhalation remains controversial. If NPs cannot penetrate the skin, they will be unable to circulate in the bloodstream, accumulate in the body, or cause side effects, ensuring their safety. Therefore, this study aimed to modify TiO_2_ NP surfaces to inhibit their uptake in skin cells. Inspired by protein corona studies, bovine serum albumin (BSA) was chosen to functionalize TiO_2_ NP surfaces via physical adsorption. The maximum BSA adsorption occurred at pH 5.0. The physicochemical properties (size, ζ-potential, morphology, ultraviolet (UV) absorption efficiency, and sun protection factor (SPF)) of TiO_2_–BSA NPs were comparable to those of TiO_2_ NPs, indicating that these properties did not affect cellular uptake. In the safety evaluation, TiO_2_ NPs and TiO_2_–BSA NPs exhibited high biocompatibility with skin cells and no phototoxicity after UVA and UVB irradiation. In the efficacy evaluation, both NPs possessed the same photoprotection abilities, reducing membrane damage and DNA breakage after UVA irradiation. Compared with TiO_2_ NPs, TiO_2_–BSA NPs showed substantially reduced skin penetration in Franz diffusion cells (91%) and human immortalized keratinocyte (HaCaT) cells (89%). A qualitative cellular uptake study using transmission electron microscopy and confocal laser scanning microscopy confirmed that TiO_2_ NPs were more abundant than TiO_2_–BSA NPs inside the HaCaT cells. These findings indicate that TiO_2_ surface functionalization with BSA inhibits cellular uptake in skin cells while maintaining safety and UV protection efficacy, which might be extended to other NP-based sunscreens.

## 1 Introduction

Ultraviolet (UV) rays released from the sun can be categorized according to their wavelengths as UVA (320–400 nm), UVB (290–320 nm), and UVC (100–290 nm). Fortunately, atmospheric ozone absorbs UVC, which is the shortest and most dangerous wavelength. UVB attacks the top layer of skin, causing redness, burning, and, with prolonged exposure, skin cancer. UVA, the longest wavelength, penetrates deeper into the dermal layer of the skin than UVB, which is the primary cause of skin aging and wrinkles. UVA exposure can stimulate reactive oxygen species (ROS) production, followed by the activation of matrix metalloproteinases (MMPs), which are proteolytic enzymes that degrade various proteins in the extracellular matrix, particularly collagen, resulting in a loss of skin elasticity.^1^

Sunscreens are therefore needed to protect skin from UVA and UVB. The most common inorganic ingredients found in effective and commercially available UV filters are zinc oxide (ZnO) and titanium dioxide (TiO_2_).^2^ These minerals have been widely used in cosmetic products, not only as UV absorbers but also as colorants, according to the European Commission database for information on cosmetic substances and ingredients (CosIng). Recently, the size of actives used in cosmetic formulations has tended to decrease from microsize to nanosize, corresponding to new findings related to their light scattering and UV protection properties. Nanosized particles scatter less light than microsized particles, producing a more transparent result that consumers prefer over opaque creams or lotions. Reinosa et al. reported that the UV protection efficiency of nanosized TiO_2_ (19 nm) was higher than that of microsized TiO_2_ (1.6 μm). However, in the case of ZnO, the micrometric size (1.4 μm) provided higher sun protection factor (SPF) values than the nanometric size (11 nm). ZnO possesses UV protection properties, against UVA, whereas TiO_2_ has an anti-UV effect against UVB. Therefore, to maintain both transparency and broad-range UV filter properties, sunscreen products should have a combination of microsized ZnO particles and nanosized TiO_2_.^3^ Moreover, ZnO is “generally recognized as safe” by the United States Food and Drug Administration when used as a UV filter according to cosmetics directives.^4^ It is generally accepted that small-sized particles, especially <150 nm, can penetrate cells;^5−7^ therefore, the safety aspects of TiO_2_ require careful consideration.

Many studies have evaluated the penetration profile and potential toxicity of TiO_2_ NPs at different levels both in vitro and in vivo using diverse methods. Crosera et al. investigated the penetration of TiO_2_ NPs (38 nm) in a synthetic sweat solution applied to Franz cells in an in vitro diffusion system for 24 h. The study demonstrated that TiO_2_ NPs did not penetrate intact or needle-damaged human skin, accumulating only in the stratum corneum and epidermis. Furthermore, low toxicity of the TiO_2_ NPs was observed after long-term exposure (7 days) to human immortalized keratinocyte (HaCaT) skin cells.^8^ Wright et al. found that the treatment of HaCaT with three different sizes and forms of TiO_2_ (<5 μm composed of 100% rutile, 21 nm composed of 80% anatase and 20% rutile, and 12 nm composed of 100% anatase) gave rise to an increase in superoxide production, caspase 8 and 9 activity, and apoptosis in a dose-dependent manner. However, there was no consistent effect on the cell viability and proliferation.^9^ Wu et al. demonstrated that TiO_2_ NPs in the range of 4–90 nm did not penetrate the stratum corneum of isolated porcine skin after in vitro exposure for 24 h in a modified Franz device. However, after 30 days of in vivo exposure to pig ear, 4 and 60 nm TiO_2_ NPs were found in a deep layer of the epidermis. Moreover, after 60 days of in vivo treatment on the dorsal skin of hairless mice, TiO_2_ NPs penetrated the skin, entered various tissues, and induced tissue damage, particularly in the skin and liver. The study suggested that after a long period of skin exposure to TiO_2_ NPs, dermal toxicity stimulation could occur, possibly associated with ROS production, oxidative stress, and collagen reduction, which are major causes of skin aging.^10^

Much evidence has shown that TiO_2_ NPs cannot penetrate the skin; however, long-term exposure to TiO_2_ NPs remains a safety concern. At ambient temperature, titanium (Ti) is highly corrosion resistant due to the formation of a thin and stable protective oxide layer on its surface.^11^ Therefore, once TiO_2_ NPs enter the body, they are difficult to degrade and eliminate, potentially causing subsequent cell and tissue damage. The effect of Ti particles on the function and morphology of immune cells was investigated. Shanbhag et al. treated macrophages with Ti particles ranging in size from 0.15 to 1.76 μm, which were smaller than the cells.^12^ They found that the release of interleukin and PGE-2 from the cells was related to particle size. Kumazawa et al. evaluated the effect of different sizes of Ti particles on the cytokine production of neutrophils. They reported that the neutrophils (5 μm) released superoxide anions and TNF-α after phagocytizing the smaller (1–3 μm) Ti particles. These secretion molecules are factors that can subsequently stimulate phagocytosis and inflammation pathways. In the case of the larger (10 μm) Ti particles, these activated cytokines were not found. This suggests that particles smaller than cells, particularly immune cells, can be phagocytized by the cells, triggering an inflammatory response.^11^

This study aims to address major concerns about TiO_2_ NP penetration through the skin by functionalizing TiO_2_ NP surfaces to inhibit skin uptake at the cellular level. According to protein corona studies of NPs used in medical applications, serum albumin, the most abundant protein in the bloodstream, coats the surface of many types of NPs, altering their physicochemical properties and identity, which can lead to a decreased level of cellular uptake in specific cell types. Human serum albumin corona on AuNPs showed a decrease in human epidermal keratinocyte uptake.^13^ Therefore, in this study, bovine serum albumin (BSA) was chosen to attach to the TiO_2_ NP surfaces via physical adsorption. The protein attachment conditions were optimized by varying the pH and the condition giving the maximum BSA adsorption was applied. Then, the physicochemical properties of the obtained particles (TiO_2_–BSA NPs), including size, ζ-potential, morphology, UV absorption efficiency, and SPF capability, were analyzed and compared to those of the unfunctionalized particles (TiO_2_ NPs). Furthermore, both NPs were investigated in terms of their toxicity and efficacy in skin cells. Finally, in vitro skin penetration and cellular uptake were studied quantitatively using inductively coupled plasma mass spectrometry (ICP–MS) in Franz diffusion cells and HaCaT cells and qualitatively using transmission electron microscopy (TEM) and confocal laser scanning microscopy (CLSM). It is well known that BSA coated on NP surfaces inhibits cell uptake. However, this is the first time that this knowledge has been applied to sunscreen-based NPs to inhibit skin cell uptake for safety reasons. The findings can be applied to NP-based sunscreen products to reduce their internalization for consumer safety.

## 2 Materials and Methods

### 2.1 Materials

TiO_2_ nanopowder with a primary particle size of 21 nm (as determined using TEM) and surface area of 35–65 m^2^/g (as determined using Brunauer, Emmett, and Teller) (product number 718467; synonyms: titania, titanium(IV) oxide, Aeroxide P25) and BSA (product number A7906) were purchased from Sigma-Aldrich. Octyl methoxycinnamate (OMC) and the base cream formulation were purchased from S. Tong Chemicals Co., Ltd. (Thailand) and Pix Botanic Co., Ltd. (Thailand), respectively. A Strat-M membrane (transdermal diffusion test model, 25 mm) was purchased from Merck (Germany). Deionized (DI) water was used throughout the work.

### 2.2 Physical Adsorption of BSA on TiO_2_ NP Surfaces

TiO_2_ NPs were dispersed in deionized (DI) water (10 mg/mL, 0.1 mL). They were added to a tube containing BSA solution diluted in DI water (5 mg/mL, 0.1 mL) and different buffer solutions (0.01 M) with various pHs from 3 to 8 (sodium acetate buffer used for pH 3–4.5 and sodium phosphate buffer used for pH 5–8) in a total reaction volume of 1 mL. The mixture was incubated at 37 °C for 3 h with shaking (300 rpm) in a thermomixer. The TiO_2_–BSA NPs were washed 3 times with Dulbecco’s phosphate-buffered saline (D-PBS) solution (pH 7.4) with centrifugation at 18,000 g and 4 °C for 30 min to remove the unbound protein. The washed TiO_2_–BSA NPs were then redispersed in D-PBS (0.1 mL) to obtain a final concentration of 10 mg/mL and maintained at 4 °C until used. The protein concentration of the supernatant was determined using a Bio-Rad protein assay according to the manufacturer’s protocol, and the absorbance was measured at 595 nm using a microplate reader (PowerWave XS2, BioTek). The protein concentration was then determined, and the amount of BSA adsorbed onto the TiO_2_–BSA NPs (Γ_ads_) at different pHs was calculated according to eq 1.
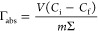
1where *V* is the solution volume (mL), *C*_i_ and *C*_f_ are the initial and equilibrium protein concentrations in the solution, respectively (mg/mL), *m* is the mass of TiO_2_ NPs (g), and Σ is the specific surface area of the TiO_2_ NPs (m^2^/g).

### 2.3 Characterization of TiO_2_ NPs

The physicochemical properties, including morphology, particle size, and polydispersity index (PDI), of the TiO_2_ NPs and TiO_2_–BSA NPs were investigated by using TEM (Jeol, JEM-2100). The particles were dropped on a carbon-coated copper grid and dried at room temperature. The PDI of the particles was analyzed from the TEM images (*n* = 100) using image processing software (ImageJ, 1.53c) and then calculated using eq 2.^14^
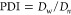
2where *D*_w_ is the weight average of the particle diameter, *D*_w_ = ∑*n_i_D_i_*^4^/∑*n_i_D_i_*^3^,

*D_n_* is the number average of the particle diameter, *D_n_* = ∑*n_i_D_i_*/∑*n_i_*, and

*n_i_* is the number of particles with a diameter of *D_i_*.

The hydrodynamic diameter (*D*_h_) and ζ-potential measurements were performed in each pH buffer solution, culture medium, or PBS buffer (1×, pH 7.4), as specified by using dynamic light scattering (DLS) (Malvern, Nano ZS, United Kingdom). The absorbance of the particles in the region of 200–800 nm was measured using an ultraviolet–visible (UV–vis) absorption spectrophotometer (PerkinElmer, Lambda 650).

### 2.4 SPF Performance

TiO_2_ NPs and TiO_2_–BSA NPs were freeze-dried and separately mixed with the base cream formulation (5 wt %). After mixing thoroughly, the sample (50 mg) was dispersed in DI water (1 mL), loaded, and spread evenly onto the poly(methyl methacrylate) (PMMA) plate (1.3 mg/cm^2^). The loaded PMMA was left at room temperature and protected from light for 5 min. SPF measurements were performed by using an SPF analyzer (Labsphere, UV-2000S). The base cream was used as a control to demonstrate the SPF performance of the TiO_2_ NPs- and TiO_2_–BSA NPs-mixed base cream formulations.

### 2.5 Cell Culture

HaCaT skin cells were cultured in Dulbecco’s modified Eagle’s medium (DMEM, Gibco) containing 10% (v/v) fetal bovine serum (Gibco), 1% (v/v) l-glutamine (Gibco), and 1% (v/v) penicillin/streptomycin (Gibco). The cells were incubated at 37 °C in a CO_2_ incubator with 95% humidity and 5% CO_2_. To dissociate adherent cells, the cells were trypsinized with 0.25% (v/v) trypsin–ethylenediaminetetraacetic acid (Gibco). The cell pellet was obtained via centrifugation at 1500 rpm for 5 min, resuspended in DMEM complete medium, and used for further assays. Viable cells were determined by using the trypan blue exclusion method.

### 2.6 Cytotoxicity Assay

The cytotoxicities of TiO_2_ NPs and TiO_2_–BSA NPs were evaluated in HaCaT and fibroblast (BJ, ATCC CRL-2522) cells at concentrations of 80–5120 μg/mL. The cells were resuspended in DMEM complete medium, cultured in 96-well plates at 8000 cells per well for HaCaT and 10,000 cells per well for fibroblasts, and incubated at 37 °C for 24 h. The cells were then treated with the NPs and further incubated at 37 °C for 24 h. The untreated sample was used as a negative control, and 2.5% dimethyl sulfoxide (DMSO) was used as a positive control. Cell viability was determined using a resazurin-based cell viability reagent (PrestoBlue, Invitrogen) according to the manufacturer’s protocol. After addition of the reagent for 3 h, the change in absorbance of the oxidized blue resazurin to red resorufin, which corresponded to the respiration activities in living cells at 570 nm using 600 nm as the reference wavelength, was measured by using a microplate reader (Molecular Devices, SpectraMax M2). The half-maximal inhibitory concentration (IC_50_) (the particle concentration that inhibits cell growth by 50%) values were calculated by fitting the curve using nonlinear regression using the Quest Graph IC_50_ Calculator.^15^

### 2.7 UVA-Induced Phototoxicity Test

The 3T3 neutral red uptake (NRU) phototoxicity test was performed in mouse fibroblast (3T3 Balb/c fibroblasts) cells purchased from ATCC (USA) according to OECD Test Guideline 432.^16−18^ Briefly, the cells were resuspended in DMEM medium containing newborn calf serum (10% v/v), glutamine (1% v/v), and Pen/Strep (1% v/v). The cells were then cultured in two 96-well plates at 10,000 cells per well and incubated at 37 °C for 24 h. The cells were treated with eight different concentrations (0.78–100 μg/mL) in sextuplicate of TiO_2_ NPs or TiO_2_–BSA NPs prepared in Earle’s balanced salt solution (EBSS) for 1 h. The +UVA plate was irradiated with 5 J/cm^2^ of UVA using a solar simulator (UVACube 400, Dr. Hönle UV technology, Germany), whereas the −UVA plate was kept in the dark at room temperature. Subsequently, the mixture was discarded, culture medium was added, and the plates were further incubated at 37 °C for 24 h. Neutral red (NR) solution (0.1 mL), prepared by diluting 1 mg of NR in 20 mL of serum-free DMEM, was added to each well. After 3 h of incubation, the cells were washed with EBSS and 0.15 mL of desorption solution (DI water/ethanol/acetic acid = 49:50:1) was added. The plates were mixed, left for NR extraction at room temperature for 10 min, and analyzed at 540 nm using the microplate reader. The photoirritation factor (PIF) and mean photo effect (MPE) values were calculated using Phototox version 2.0 software. According to the OECD Test Guideline 432, a substance is predicted to be phototoxic if PIF ≥ 5 or MPE ≥ 0.15. A test substance with 2 ≤ PIF < 5 or 0.1 ≤ MPE < 0.15 is predicted as “probably phototoxic” and a test substance with PIF < 2 or MPE < 0.1 is predicted to have “no phototoxicity.”^19^ Chlorpromazine (CPZ) was used as a positive control. The results are the means of at least two independent experiments.

### 2.8 UVB-Induced Phototoxicity Test

The HaCaT cells were resuspended in DMEM complete medium, cultured in two 96-well plates at 8000 cells per well, and incubated at 37 °C for 24 h. The cells were treated with eight different concentrations (0.78–100 μg/mL) in sextuplicate of TiO_2_ NPs or TiO_2_–BSA NPs prepared in EBSS for 1 h. The +UVB plate was irradiated with 200 mJ/cm^2^ UVB, whereas the −UVB plate was kept in the dark at room temperature. Cell viability was determined using a resazurin-based cell viability reagent (PrestoBlue, Invitrogen) according to the manufacturer’s protocol. After addition of the reagent for 3 h, the absorbance at 570 nm using 600 nm as the reference wavelength was measured using a microplate reader (PowerWave XS2, BioTek). The PIF and MPE values were calculated using Phototox version 2.0 software. The phototoxicity was predicted as mentioned above. CPZ was used as a positive control.

### 2.9 Photoprotection Ability

HaCaT cells at a density of 8000 cells were cultured in two 96-well black/clear-bottom plates and treated with TiO_2_ NPs or TiO_2_–BSA NPs at a concentration of 5 μg/mL for 1.5 h. Next, one plate was irradiated with 25 J/cm^2^ of UVA using a solar simulator (UVACube 400, Dr. Hönle UV technology, Germany), and another plate was kept in the dark. Then, the mixture was removed and DMEM complete medium was added. After 24 h of incubation, the membrane protection ability was evaluated by replacing the old medium with Image-IT DEAD Green Viability Stain (Invitrogen) diluted in DMEM complete medium. According to the manufacturer’s instructions, the cells were stained under normal cell culture conditions for 30 min, fixed with 4% (v/v) paraformaldehyde in PBS solution at room temperature for 15 min, permeabilized with 0.1% (v/v) Triton X-100 at room temperature for 15 min, and blocked in 1% (w/v) BSA solution at room temperature for 1 h. To investigate the effect of UV exposure on DNA damage, antiphospho-H2A.X (Ser139), clone JBW30, and Alexa Fluor 555 Conjugate (Sigma-Aldrich) antibody prepared in 1% (w/v) BSA solution was added to the cells and further incubated for another hour. After washing and adding D-PBS buffer, the fluorescence intensity was measured using a microplate reader (Synergy H1, BioTek) with excitation at 488 nm and emission at 515 nm for Image-IT DEAD Green detection and excitation at 555 nm and emission at 580 nm for antiphospho-H2A.X (Ser139) detection. The fluorescence intensity was normalized by comparing untreated and nonirradiated cells.

### 2.10 Qualitative Cellular Uptake Study Using TEM

To track the beginning of the uptake process, a 24-well plate was used to culture 100,000 HaCaT cells resuspended in DMEM complete medium for 24 h. Then, the cells were incubated with 20 μg/mL TiO_2_ NPs or TiO_2_–BSA NPs prepared in serum-free DMEM for 3 h. Next, the cells were washed twice, trypsinized, and collected in a 1.5 mL tube. Then, primary fixation was performed using 5% (v/v) glutaraldehyde diluted in D-PBS and incubated at 4 °C for 12 h. The cell pellet was washed three times with D-PBS for 10 min. Subsequently, secondary fixation was performed at room temperature for 2 h using 1% (v/v) osmium tetroxide in distilled water. After washing 3 times with distilled water for 10 min, the cells were dehydrated in a series of acetone concentrations diluted in distilled water (10, 30, 50, 70, 90%, and 3 times at 100% v/v) for 10 min followed by infiltration with acetone/Spurr’s resin (1:1, 1:2, and 2:1 volume ratios) for 3–4 h and pure Spurr’s resin three times for 3–4 h. The prepared sample was embedded in an embedding mold, incubated at 80 °C for 7 h, and cut using an ultramicrotome (UC7, Leica, Germany). Finally, an ultrathin section with a thickness of 70 nm was placed on top of the copper grid, stained with 5% (v/v) aqueous uranyl acetate for 15 min and lead citrate for 15 min, and observed using TEM (HT7700, Hitachi, Japan).

### 2.11 Qualitative Cellular Uptake Study Using CLSM

HaCaT cells were resuspended in DMEM complete medium and seeded in a 35 mm Nunc glass-bottom dish for 24 h at a density of 75,000 cells. Then, the cells were incubated with 20 μg/mL TiO_2_ NPs or TiO_2_–BSA NPs prepared in serum-free DMEM for 24 h. Subsequently, a colocalization assay was performed to indicate cellular uptake of the TiO_2_ NPs and TiO_2_–BSA NPs. The cells were washed three times with D-PBS and stained with LysoTracker Red DND-99 dye (50 nM, Life Technologies) for 1 h to track the lysosomes. After washing with D-PBS, the cells were imaged using the Z-stack mode of a CLSM (TCS SP8 STED, Leica, Germany).

### 2.12 Quantitative Cellular Uptake Study Using ICP–MS

HaCaT cells were resuspended in DMEM complete medium (100,000 cells) and cultured in a 24-well plate for 24 h. Then, the cells were further incubated with 20 μg/mL TiO_2_ NPs or TiO_2_–BSA NPs prepared in serum-free DMEM for 24 h. Next, the cells were washed twice, trypsinized, collected in a 1.5 mL tube, and resuspended in D-PBS. The samples were subjected to digestion using a microwave digester (Ultrawave, Milestone) and analyzed for Ti using ICP–MS (ICPMS-2030, Shimadzu, Japan) at the NSTDA Characterization and Testing Service Center (Thailand). The amount of detected Ti was reported as the cumulative Ti (μg) in HaCaT cells.

### 2.13 Evaluation of Transdermal Ability

Skin penetration of TiO_2_ NPs and TiO_2_–BSA NPs was evaluated in a synthetic Strat-M membrane (Merck), a nonanimal-based model for transdermal diffusion testing using the Franz diffusion cells approach. The donor chamber of the Franz cell was loaded with TiO_2_ NP or TiO_2_–BSA NPs diluted in D-PBS (1 mg/mL, 1 mL), whereas the receptor chamber was filled with D-PBS. The system was connected to a temperature-controlled circulating water bath at 32.5 °C with a stirring rate of 500 rpm. The penetrated samples were collected from the receptor chamber at 0, 30 min, and 2, 4, 8, 12, and 24 h. The samples were subjected to digestion and analyzed for Ti using ICP–MS as mentioned above. The amount of Ti detected was reported as cumulative Ti (μg) after passing through a Strat-M membrane.

## 3 Results and Discussion

### 3.1 Physical Adsorption of BSA on TiO_2_ NP Surfaces

**Figure 1:**
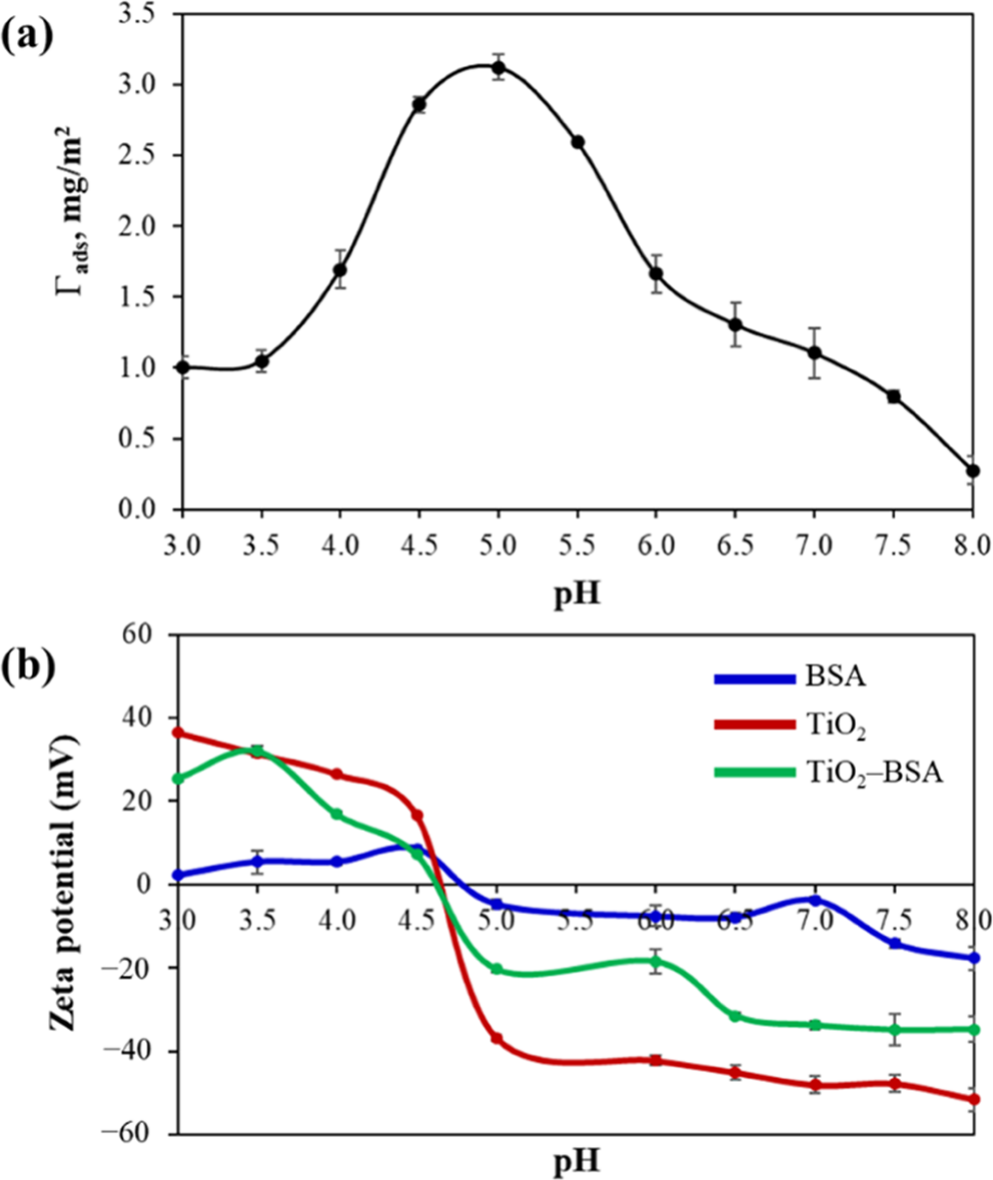
Adsorption of BSA on TiO_2_ NP surfaces. (a) The amount of BSA adsorbed onto the TiO_2_–BSA NPs (Γ_ads_) at different pHs. (b) ζ-potential values of BSA, TiO_2_ NPs, and TiO_2_–BSA NPs at different pHs.

A physical adsorption approach of BSA was selected to minimize the change in the surface properties of the NPs. TiO_2_ NPs are currently listed in the European Commission CosIng database, indicating that they can be used in cosmetic products. When their surface properties are altered, the altered TiO_2_ NPs are categorized as a “new material.” They must be tested for any potential toxicity such as genotoxicity, phototoxicity, acute toxicity, skin irritation, skin sensitization, and ocular irritation. In addition, BSA is on the CosIng list, making it suitable for the modification of NPs used in cosmetics, such as in this study. Many parameters affect protein attachment on solid substrates, including pH, ionic strength, protein concentration, incubation time, and surface chemistry.^20^ Therefore, suitable conditions for each protein–NP adsorption system require optimization. To investigate the effect of pH, the ionic strength of the adsorption buffer was adjusted to as low as 0.01 M while other parameters were fixed. Physical adsorption of BSA on TiO_2_ NP surfaces was performed at pHs ranging from 3.0 to 8.0 and incubated at 37 °C for 3 h. After the separation of the NPs via centrifugation, the unbound protein was measured, and the amount of BSA adsorbed onto the TiO_2_ NPs (Γ_ads_) at different pHs was calculated (Figure 1a). The results showed that the BSA adsorption behavior on the TiO_2_ NP surfaces was strongly pH-dependent. The maximum Γ_ads_ (3.122 ± 0.093 mg/m^2^) occurred at pH 5.0, which is close to the isoelectric point (pI) of BSA (4.7). At pH values below and above 5.0 (pH 3.0–4.0 and 6.0–8.0, respectively), Γ_ads_ tended to decrease dramatically. This adsorption behavior has been observed in previous studies using different types of NPs.^21−23^ The attractive surface charges between the protein and the NPs and the conformational change of the protein at a given pH contribute to the physical adsorption process.^21−24^ Therefore, the ζ-potential values of BSA and the NPs were determined at different pH values to explain the surface charges involved in the adsorption behavior (Figure 1b). At a pH near the pI of BSA (the ζ-potential value = 0), the BSA conformation mainly appeared as the helix state (55%), producing the most compact molecule, which gave rise to the highest Γ_ads_ at this point. At pH values below or above the pI of BSA, both BSA and TiO_2_ NPs carried the same charges. Thus, at pH < 4.7, BSA and the TiO_2_ NPs possessed positive surface charges, whereas at pH > 4.7, BSA and the TiO_2_ NPs expressed negative surface charges. The decrease in Γ_ads_ in these ranges resulted from the strong repulsive electrostatic interactions, which were mainly attributed to repellent forces among BSA molecules and between BSA and the NPs.^20^ Furthermore, the obtained TiO_2_–BSA NPs expressed ζ-potential values that were more negative than those of the TiO_2_ NPs across all pH ranges evaluated due to the negativity of the BSA molecule, confirming the attachment of BSA on the particle surfaces. Therefore, the adsorption buffer at pH 5.0 producing the maximum Γ_ads_ was chosen as the optimal condition for physically attaching BSA to the TiO_2_ NP surfaces and preparing the particles for further study.

### 3.2 Characterization of TiO_2_ NPs

**Figure 2:**
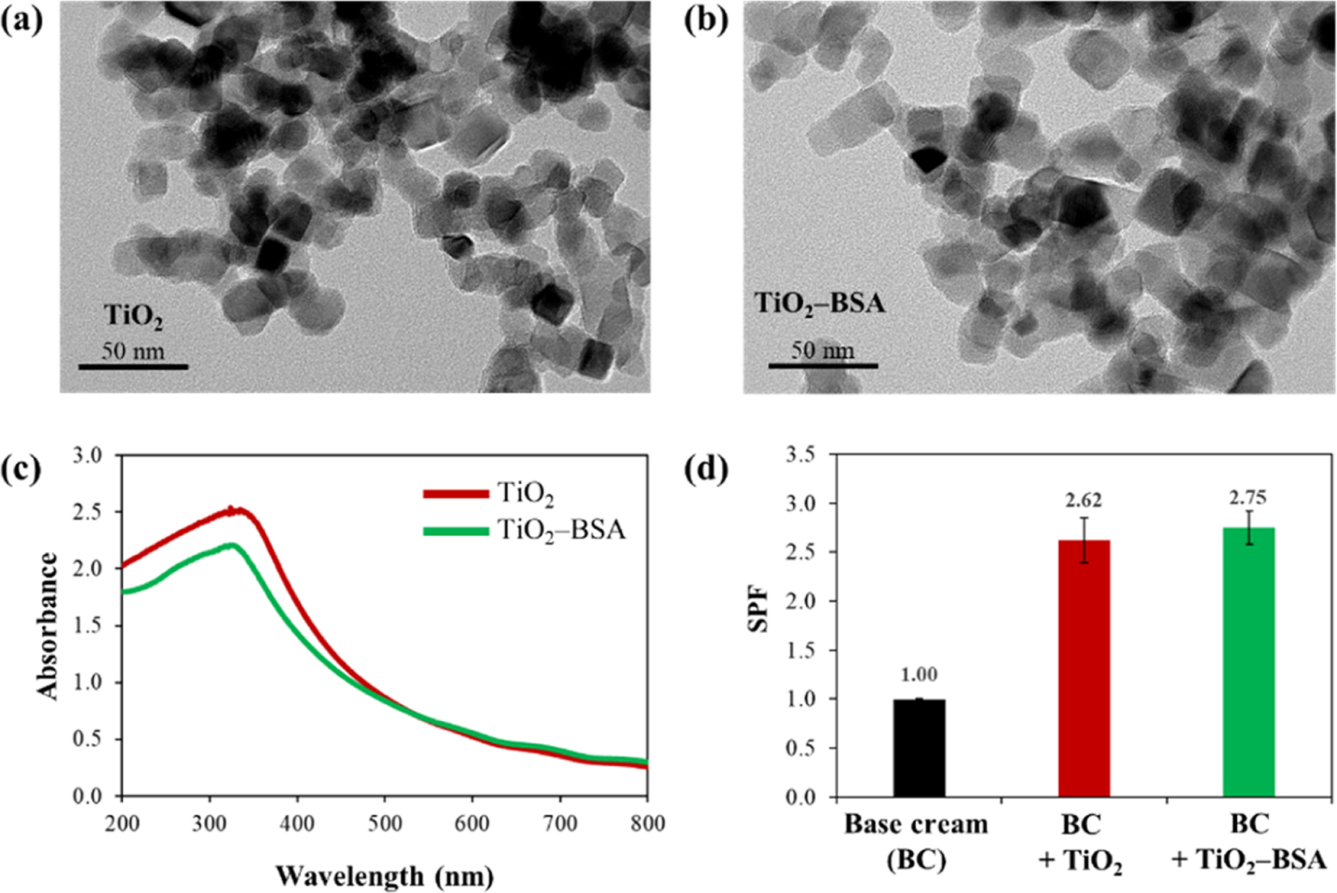
Characterization of TiO_2_ NPs and TiO_2_–BSA NPs. TEM micrographs of (a) TiO_2_ NPs and (b) TiO_2_–BSA NPs. (c) Absorbance spectra and (d) SPF values.

The TiO_2_ NPs and the TiO_2_–BSA NPs were observed using TEM. As shown in Table 1 and Figure 2a,b, both NPs had irregular shapes and were quite similar in primary size (20.52 ± 4.40 nm for the TiO_2_ NPs and 22.88 ± 5.79 nm for the TiO_2_–BSA NPs). The PDI values determined from the TEM images were >1.05, indicating a slightly broad size distribution of the samples.^25^Figure S1(a,b) shows typical crystal lattice fringes of the TiO_2_ NPs at high magnification of the TEM micrographs. A gelatinous layer of BSA was observed on the TiO_2_–BSA NPs (Figure S2(a,b)), confirming the BSA coating on the surface.

**Table 1:** Size, PDI, and ζ-Potential Values of TiO_2_ NPs and TiO_2_–BSA NPs

measurement		TiO_2_	TiO_2_–BSA
dried state using TEM	*D_n_* (nm)	20.52 ± 4.40	22.88 ± 5.79
	PDI	1.14	1.25
hydrodynamic state in PBS using DLS	*D*_h_ (nm)	1,829.67 ± 41.93	928.30 ± 72.75
	PDI	0.14 ± 0.12	0.19 ± 0.05
	ζ-potential (mV)	–27.00 ± 1.78	–24.54 ± 1.51
hydrodynamic state in DMEM using DLS	*D*_h_ (nm)	1,870.67 ± 92.41	1,285.67 ± 25.98
	PDI	0.52 ± 0.08	0.15 ± 0.045
	ζ-potential (mV)	–11.54 ± 0.21	–11.78 ± 0.74

To understand the surface charge expression during incubation with the cells, the ζ-potential values of the particles diluted in serum-free cell culture medium (DMEM) were measured and showed the same negative surface charges. Therefore, under the cell culture conditions, the surface charges were the same and were not affected by a difference in their cellular uptake efficiency. The TiO_2_ NPs diluted in PBS buffer (1×, pH 7.4) showed charges that were slightly more negative than those of the TiO_2_–BSA NPs. The *D*_h_ values of the TiO_2_ NPs diluted in serum-free DMEM and PBS buffer were greater than those of the TiO_2_–BSA NPs. This is probably because, at pH 7.4 of the medium, the globular BSA acted as a stabilizing layer by creating a steric repulsive force between the TiO_2_–BSA NPs.^26^ A reduction in the aggregate size of the NPs after protein adsorption has been observed in various types of NPs such as TiO_2_ NPs, fullerenes, polystyrene, magnetite, tungsten carbide, ceria oxide, and zirconia NPs.^27^

Furthermore, the TiO_2_–BSA NPs exhibited a broad range of absorbance spectra, which is a typical characteristic of TiO_2_ NPs,^28^ and showed similar SPF values to the TiO_2_ NPs (Figure 2c,d). These results demonstrate that the physical adsorption of BSA on TiO_2_ NP surfaces did not affect their physicochemical properties, including size, shape, absorbance pattern, and SPF performance. This indicates that the TiO_2_–BSA NPs obtained using this method can be used in cosmetics in the same manner as the TiO_2_ NPs listed in the CosIng database according to the regulations.

### 3.3 Cytotoxicity Assay

**Figure 3:**
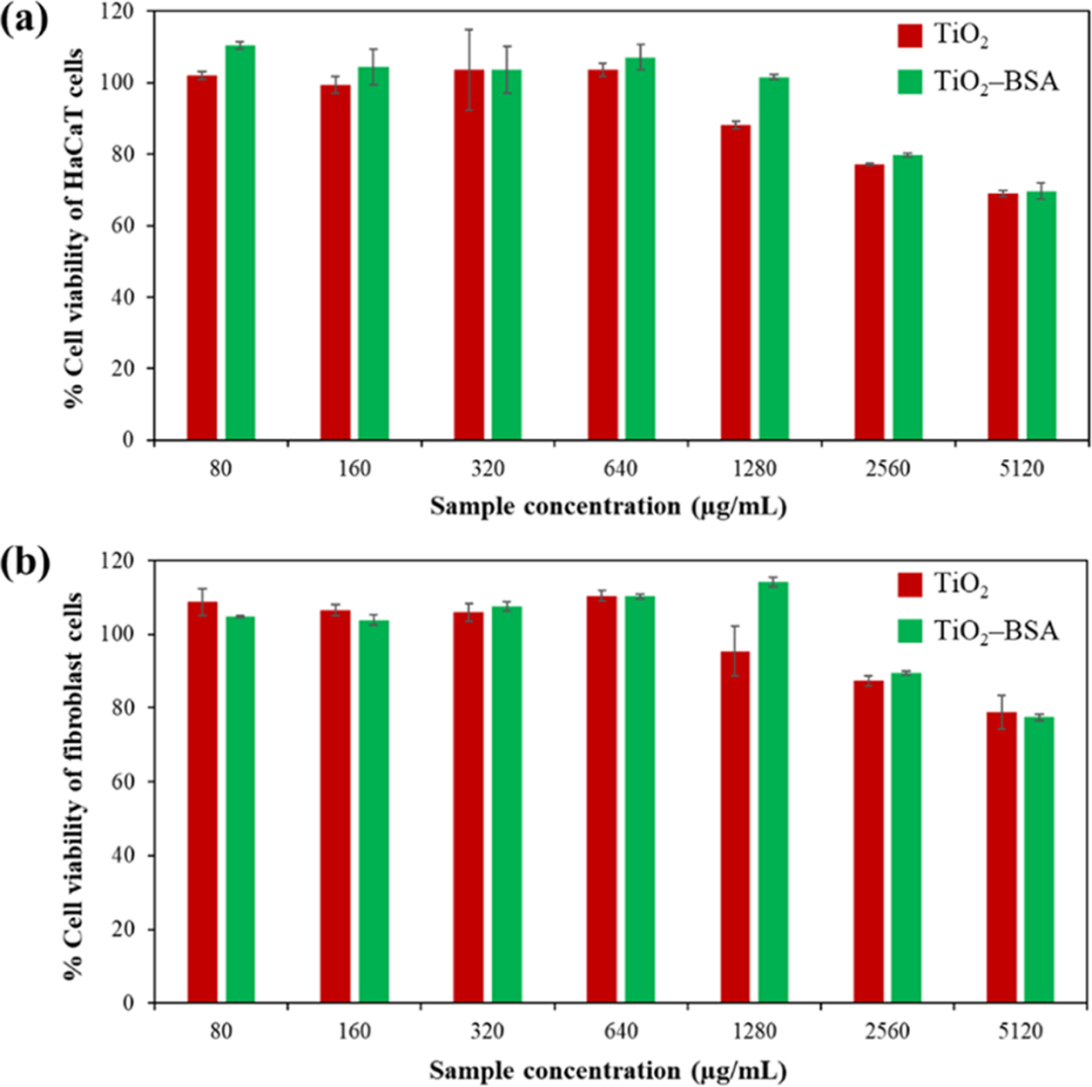
Cytotoxicity evaluation of TiO_2_ NPs and TiO_2_–BSA NPs after 24 h of incubation in human skin: (a) HaCaT and (b) fibroblast cells.

Aside from the physicochemical properties, TiO_2_ NPs and TiO_2_–BSA NPs were evaluated for their safety in two types of human skin cell lines: keratinocytes (HaCaT) and fibroblast cells. The TiO_2_ NPs and TiO_2_–BSA NPs were incubated with the cells under normal cell culture conditions for 24 h. Cell viability was then determined using a resazurin-based cell viability reagent. The absorbance was measured as the oxidized blue resazurin turned to red resorufin in proportion to the metabolic activities of living cells. The untreated control cells were calculated as 100% cell viability. The cell viability percentages of the treated HaCaTs and fibroblasts were calculated (Figure 3a,b). Dose-dependent toxicity was observed at very high particle concentrations. At a total particle concentration of 1280 μg/mL, the viabilities of both cell types were >80%. The TiO_2_ NPs and the TiO_2_–BSA NPs exhibited high IC_50_ values of 9.606 × 10^3^ and 1.275 × 10^4^ μg/mL, respectively, for the HaCaT cells and 8.346 × 10^3^ and 8.623 × 10^3^ μg/mL, respectively, for the fibroblast cells, indicating excellent biocompatibility of these particles.

### 3.4 Phototoxicity Test

In sunlight, UVA and UVB are responsible for phototoxicity. UVA penetrates deeper into the subcutaneous layer than UVB, reaching capillary blood and activating ROS production, which induces DNA breakage. Fortunately, UVB travels only to the stratum corneum of the epidermal layer. Moreover, energy loss of UVB occurs during its passage through the atmosphere, resulting in an energy level that is 100-fold less than that of UVA at sea level.^29,30^ Therefore, the phototoxic potential of a test chemical against UVB is considered to be less important than that against UVA for systemic formulations. However, UVB-induced phototoxicity is related to topical formulations, especially UVB filter substances.^31^

The in vitro 3T3 NRU phototoxicity test is used to identify the phototoxic potential of a tested substance activated by UVA exposure. The assay was performed following OECD Test Guideline 432. After treatment of BALB/3T3 mouse embryonic fibroblasts with the NP samples and irradiation, cell viability was evaluated by using NR dye, which penetrates and accumulates in the lysosomes of viable cells. The UVB-induced phototoxicity test was performed using the epidermal HaCaT cells via modification using the NRU phototoxicity test. The Phototox version 2.0 software was used to calculate the PIF and MPE values in both assays (Table 2). No potential phototoxicity was observed from TiO_2_ NPs or TiO_2_–BSA NPs after UVA and UVB irradiation, indicating their safety as actives in sunscreen products. Conversely, the positive control (CPZ) showed phototoxicity after UVA and UVB irradiation. For the UVA phototoxicity test, the PIF and MPE values of CPZ corresponded to the test acceptance criteria (PIF > 14.4 and 0.33 ≤ MPE ≤ 0.63). For the UVB phototoxicity test, for which there was no guideline, the cell viability of CPZ-treated HaCaT cells at 12.5 μg/mL was 30.8 ± 11.7%, which was close to the results obtained from the CPZ-treated L-929 cells (56.90 ± 8.05%) and CPZ-treated NIH-3T3 cells (48.6 ± 3.37%) at 10 μg/mL.^32^

**Table 2:** Calculated PIF and MPE Values of TiO_2_ NPs, TiO_2_–BSA NPs, and CPZ

	UVA irradiation			UVB irradiation		
sample	PIF	MPE	interpretation	PIF	MPE	interpretation
TiO_2_	1.0	0.042	no phototoxicity	1.0	–0.059	no phototoxicity
TiO_2_–BSA	1.0	0.012	no phototoxicity	1.0	–0.007	no phototoxicity
CPZ	23.22	0.56	phototoxicity	7.41	0.18	phototoxicity

### 3.5 Photoprotection Ability

**Figure 4:**
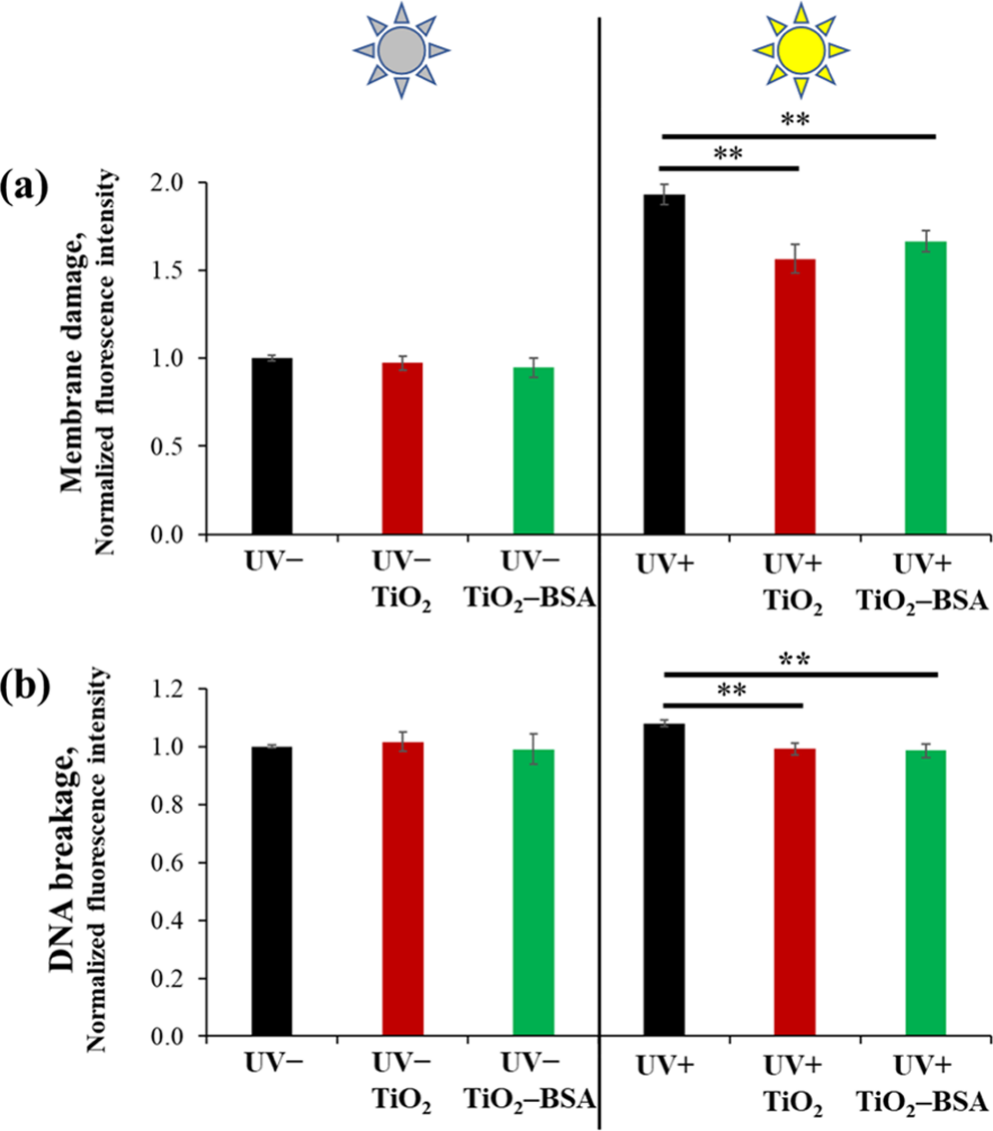
Photoprotection ability evaluation of TiO_2_ NPs and TiO_2_–BSA NPs after exposure to UVA in terms of (a) membrane damage and (b) DNA breakage.

UV exposure can cause cellular damage, resulting in cellular malfunctions and death.^33^ In this work, we evaluated the photoprotection abilities of TiO_2_ NPs and TiO_2_–BSA NPs for membranes and DNA molecules. The TiO_2_ NPs and TiO_2_–BSA NPs were incubated with HaCaT cells prior to UVA exposure to simulate the application of a sunscreen product. After 24 h of recovery time, Image-IT DEAD Green Viability Stain was used to investigate the membrane integrity. This fluorophore cannot penetrate the cell membranes of healthy cells. Once the membrane is compromised, it is permeable to the dye, suggesting that those membranes are injured. Later, Alexa Fluor 555 conjugated antiphospho-H2A.X antibody was added to the cells to detect strand DNA damage. When double-stranded DNA breaks, a histone protein (H2A.X) is rapidly phosphorylated at serine 139. This phosphorylation process can be amplified, and finally, the damaged area is indicated, resulting in the recruitment of several proteins involved in the DNA repair step. Therefore, a high expression of phosphorylated H2A.X can be used as a signal for DNA breakage. The results showed that the untreated cells suffered from UVA irradiation, which caused greater membrane damage and DNA breakage (Figure 4a,b) compared to nonirradiated cells. In contrast, the cells treated with TiO_2_ NPs and TiO_2_–BSA NPs showed substantially less membrane damage and DNA breakage than irradiated and untreated cells. Notably, the membrane damage and DNA breakage signals of TiO_2_ NP- and TiO_2_–BSA NP-treated and irradiated cells were not different. This suggests that the photoprotection ability of TiO_2_–BSA NPs was similar to that of TiO_2_ NPs, indicating that the BSA coating had no effect on the UV protection properties.

### 3.6 Qualitative Cellular Uptake Study Using TEM

**Figure 5:**
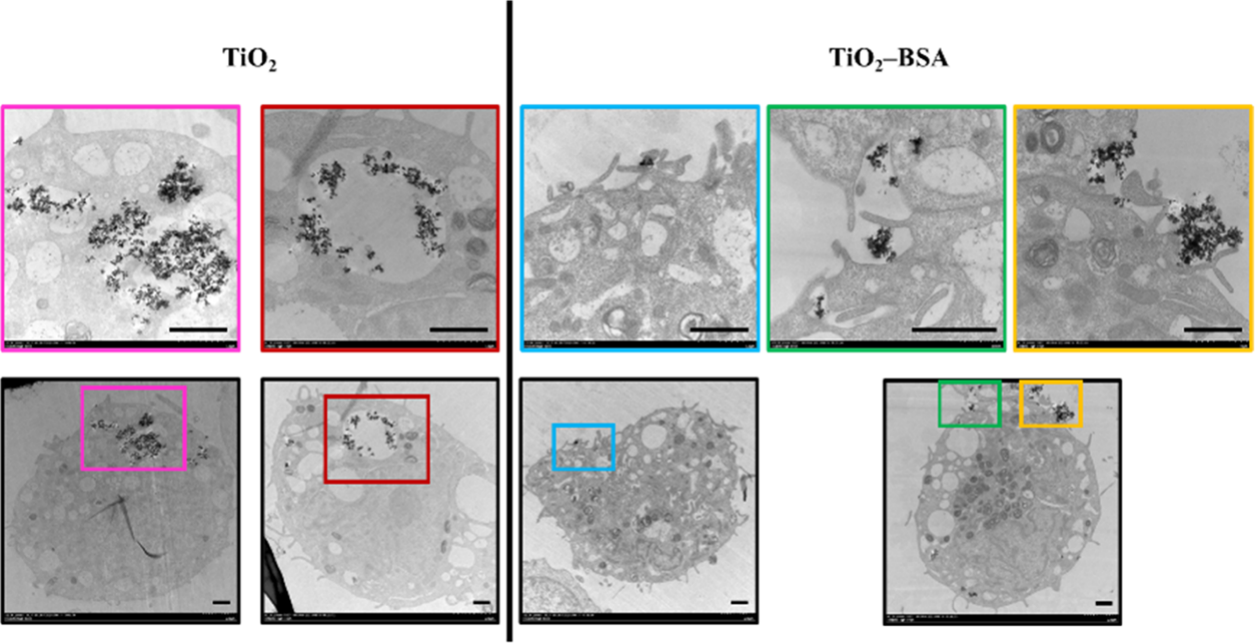
Qualitative cellular uptake study using TEM of TiO_2_ NPs and TiO_2_–BSA NPs after incubation in HaCaT cells for 3 h. The upper row shows the magnified areas from the corresponding photos in the lower row, indicated by the same colored borders. The scale bar is 1 μm.

The safety and UV protection efficacy of TiO_2_–BSA NPs were demonstrated, as mentioned above. Next, cellular uptake, which is the key result of this study, was investigated using several techniques, including qualitative methods, such as TEM and CLSM, and quantitative methods, such as ICP–MS. According to our hypothesis, BSA-coated TiO_2_ NPs may reduce NP absorption into skin cells. Therefore, we performed a cellular uptake study by incubating TiO_2_ NPs and TiO_2_–BSA NPs with HaCaT cells in serum-free DMEM to avoid the effects of serum protein coating and displacement on their surface during incubation.^34^ The incubation time was 3 h to observe the uptake behavior from an early step. The treated cells were fixed and observed by using TEM (Figure 5). The upper row shows magnified areas from the corresponding photos in the lower row, which are indicated by the same colored borders. After 3 h of incubation, the TEM results showed that TiO_2_ NPs were abundant inside the HaCaT cells, appearing as aggregated dark dots. Conversely, TiO_2_–BSA NPs had not yet penetrated the cells, and some were observed outside the cells, indicating that the TiO_2_–BSA NPs could decelerate the uptake process. NPs can enter cells via different routes, including clathrin-mediated endocytosis, caveolin-mediated endocytosis, macropinocytosis, phagocytosis, and spontaneous translocation across the plasma membrane.^35^ Interestingly, the typical characteristics of the macropinocytosis cellular uptake pathway, which involves the formation of large (200 nm to 5 μm in diameter) membrane protrusions,^36^ was observed in the cells treated with TiO_2_–BSA NPs. The results indicate that the TiO_2_–BSA NPs may use this pathway to enter the cells. However, membrane protrusion formation was not observed in the cellular uptake of TiO_2_ NPs. Macropinocytosis, which naturally occurs in nearly all cell types, is defined as a nonselective pathway for cells to engulf extracellular fluid and its content. This means that, unlike other endocytosis pathways, loading does not require the binding of specific cellular receptors to initiate the internalization process.^37,38^ TiO_2_ NPs (Aeroxide P25) in a size range of 30–100 nm have been reported to enter fish liver parenchymal cells (RTL-W1) via the caveolae-mediated endocytosis pathway.^35^ The modification of NPs is crucial, which is affected not only by their intracellular fate but also by their entry pathway.^38^

### 3.7 Qualitative Cellular Uptake Study Using CLSM

**Figure 6:**
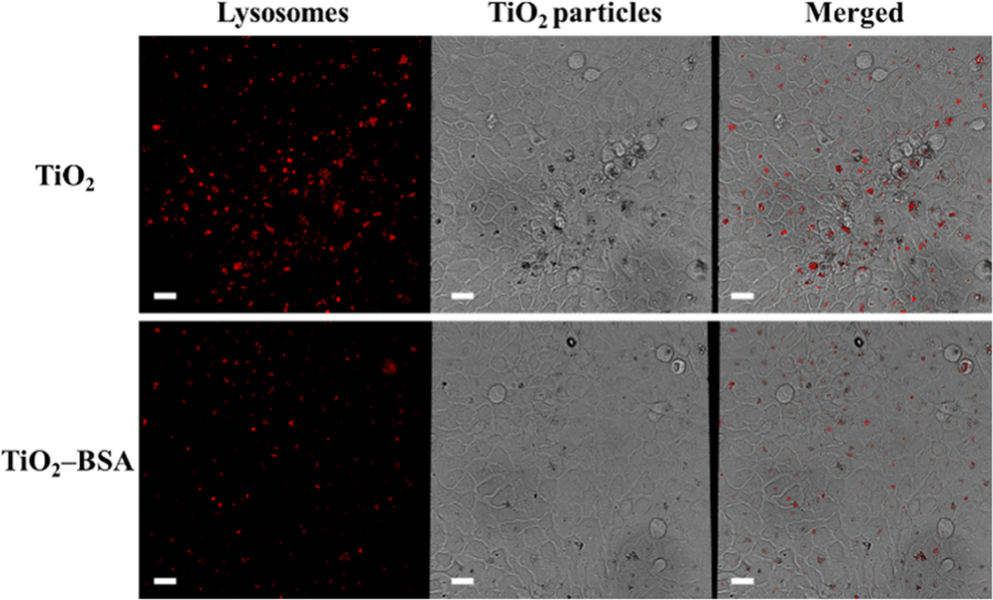
Qualitative cellular uptake study using CLSM of TiO_2_ NPs and TiO_2_–BSA NPs after incubation in HaCaT cells for 24 h. Lysosomes are shown in red. Particles are shown in black. The scale bar is 5 μm.

The uptake was monitored for 24 h, and HaCaT cells were treated with TiO_2_ NPs or TiO_2_–BSA NPs prepared in serum-free DMEM. Next, a colocalization assay was performed using LysoTracker Red DND-99 dye to track the lysosomes. As shown in Figure 6, colocalization between the lysosomes (red) and the particles (black) occurred in the merged channel. Because lysosomes are cellular organelles, this colocalization confirmed the internalization of the NPs inside the HaCaT cells. However, the colocalization of TiO_2_–BSA NPs was lower than that of TiO_2_ NPs, suggesting that TiO_2_–BSA NPs had a lower cellular uptake than TiO_2_ NPs.

### 3.8 Quantitative Cellular Uptake Study Using ICP–MS

**Figure 7:**
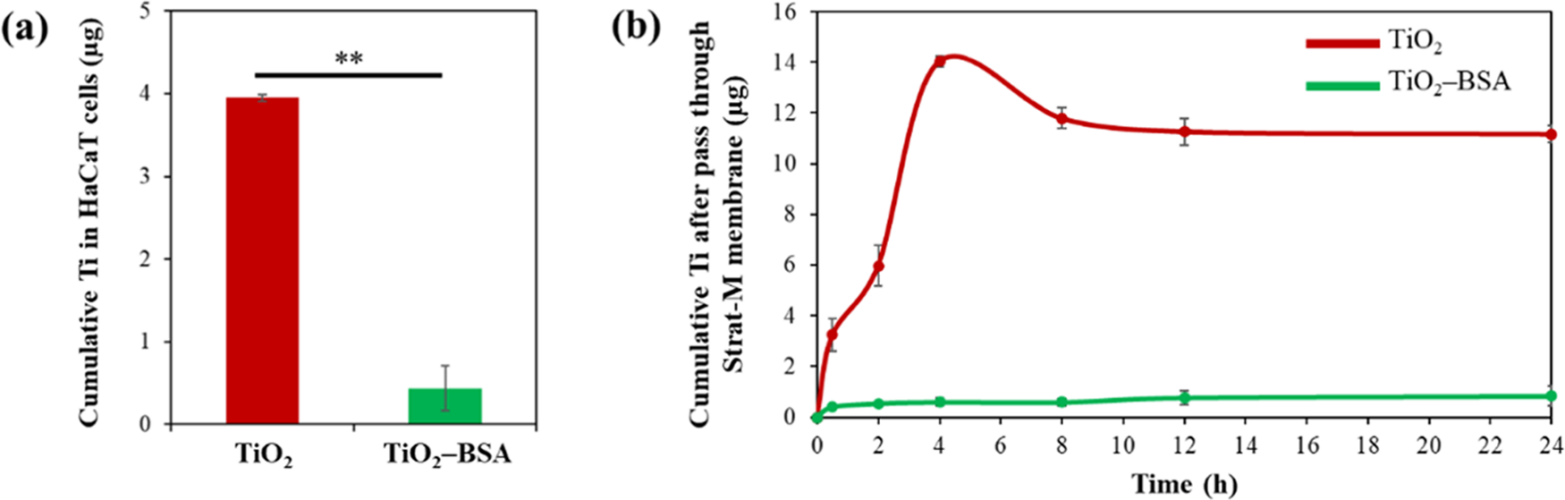
Detection of the Ti amount using ICP–MS. (a) Cellular uptake study of TiO_2_ NPs and TiO_2_–BSA NPs after incubation in HaCaT cells for 24 h. (b) Transdermal diffusion testing of TiO_2_ NPs and TiO_2_–BSA NPs evaluated in a synthetic Strat-M membrane using the Franz diffusion cells system.

Cellular uptake of TiO_2_ NPs and TiO_2_–BSA NPs in HaCaT cells was further investigated quantitatively. After 24 h of incubation, the cells were washed, harvested, and analyzed for Ti using ICP–MS. Figure 7a demonstrates a dramatic decrease (89%) in the cellular uptake of TiO_2_–BSA NPs compared to TiO_2_ NPs. This quantitative evidence supports the qualitative cellular uptake study using TEM and CLSM as mentioned previously.

### 3.9 Evaluation of Transdermal Ability

Using animal models for cosmetic purposes was banned in many countries, particularly in Europe. Therefore, in this study, we used a Strat-M membrane, a synthetic nonanimal-based model that is generally accepted as a representation of human skin in skin penetration studies. The transdermal ability of TiO_2_ NPs and TiO_2_–BSA NPs through a Strat-M membrane was performed using the Franz diffusion cells. The penetrated samples were collected from the receptor chamber at different times for 24 h. The amount of detected Ti was measured using ICP–MS and reported as the cumulative Ti after passing through a Strat-M membrane (μg) (Figure 7b). The amount of Ti in the TiO_2_ NP-penetrated samples increased rapidly within 4 h compared to that in the TiO_2_–BSA NP-penetrated samples and remained constant after 8 h. At 24 h, TiO_2_–BSA NPs showed a 92.5% decrease in the cumulative Ti after passing through the Strat-M membrane compared to TiO_2_ NPs. Surprisingly, the reduced cumulative Ti in cellular uptake was highly comparable to the results of the transdermal diffusion test. This indicates the high possibility that the BSA coating helps to prevent the internalization of TiO_2_–BSA NPs.

## 4 Conclusions

BSA was successfully physically attached to the TiO_2_ NP surfaces. The obtained TiO_2_–BSA NPs were comparable to TiO_2_ NPs in terms of their physicochemical properties, safety, and UV protection efficacy. Moreover, the TiO_2_–BSA NPs showed decreased in vitro skin penetration in Franz diffusion cell devices and reduced cellular uptake in HaCaT skin cells. These findings suggest that BSA functionalization on NP surfaces can protect skin cells from invading NPs, which can be applied in NP-based sunscreens and other NPs to reduce internalization and to ensure consumer safety. Physical adsorption, which was used to prepare the TiO_2_–BSA NPs, is a simple process with low power and chemical consumption, making it ideal for industrial-scale production.

## Supporting Information Available

TEM micrographs of TiO_2_ NPs at magnification 250,000× and 500,000×, and TEM micrographs of TiO_2_–BSA NPs at magnification 250,000× and 500,000× (PDF)
