# Supplementary material for: BSA Adsorption on Titanium Dioxide Nanoparticle Surfaces for Controlling Their Cellular Uptake in Skin Cells
Source: ACS Appl Bio Mater. 2024 Mar 4;7(3):1713–22. doi: 10.1021/acsabm.3c01138 (PMC10951944; doi:10.1021/acsabm.3c01138)
Supplement: Supplementary file 1 — mt3c01138_si_001.pdf [file mt3c01138_si_001.pdf]

Supporting Information

BSA Adsorption on Titanium Dioxide Nanoparticle Surfaces for Controlling Their Cellular Uptake in Skin Cells

Raweewan Thiramanas*, Yodsathorn Wongngam, Goragot Supanakorn, and Duangporn Polpanich

National Nanotechnology Center (NANOTEC), National Science and Technology Development Agency (NSTDA), Pathum Thani, 12120, Thailand

*Corresponding author: raweewan@nanotec.or.th

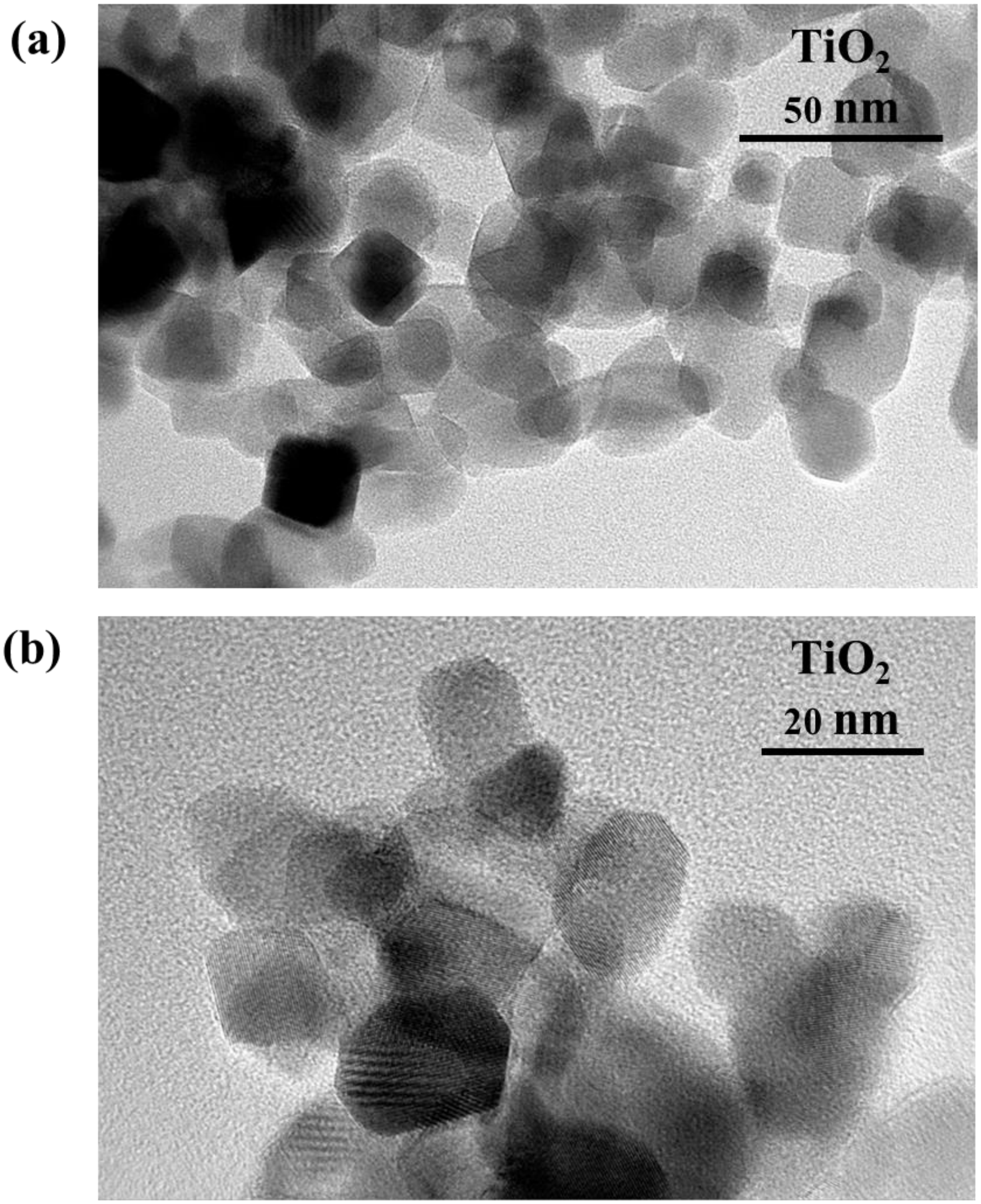

Figure S1. TEM micrographs of TiO_2 NPs at magnification 250,000× (a) and 500,000× (b).

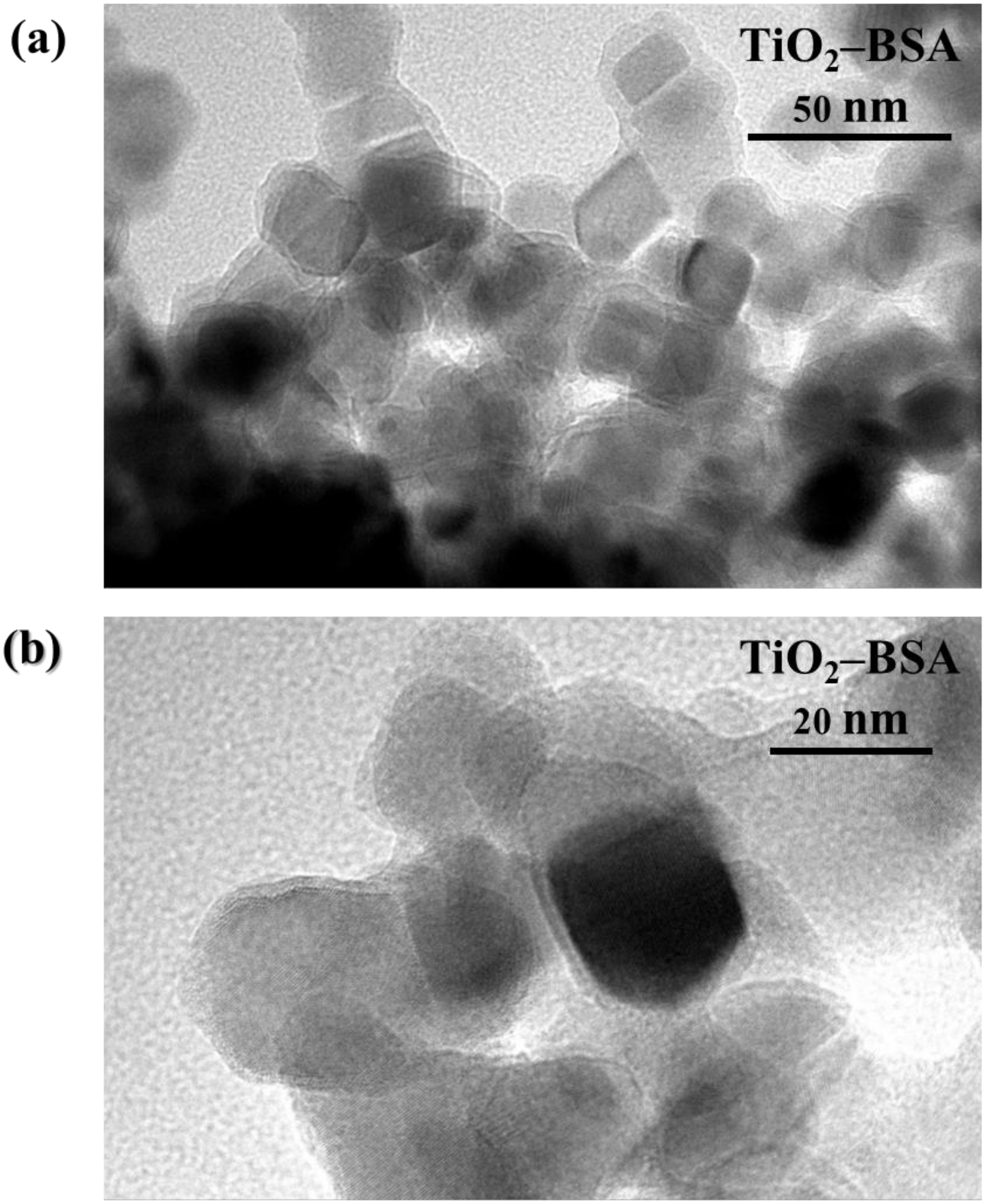

Figure S2. TEM micrographs of TiO_2–BSA NPs at magnification 250,000× (a) and 500,000× (b).
